# Chronic Intermittent Ethanol Exposure Alters Stress Effects on (3α,5α)-3-hydroxy-pregnan-20-one (3α,5α-THP) Immunolabeling of Amygdala Neurons in C57BL/6J Mice

**DOI:** 10.3389/fncel.2016.00040

**Published:** 2016-03-04

**Authors:** Antoniette M. Maldonado-Devincci, Alexander Kampov-Polevoi, Raechel E. McKinley, Danielle H. Morrow, Todd K. O’Buckley, A. Leslie Morrow

**Affiliations:** ^1^Department of Psychology, North Carolina Agricultural and Technical State UniversityGreensboro, NC, USA; ^2^Bowles Center for Alcohol Studies, University of North Carolina at Chapel HillChapel Hill, NC, USA; ^3^Department of Biology, North Carolina Agricultural and Technical State UniversityGreensboro, NC, USA; ^4^Department of Psychiatry, University of North Carolina at Chapel HillChapel Hill, NC, USA; ^5^Department of Pharmacology, University of North Carolina at Chapel HillChapel Hill, NC, USA

**Keywords:** alcohol, 3α, 5α-THP, allopregnanolone, neuroactive steroid, withdrawal, stress, mouse

## Abstract

The GABAergic neuroactive steroid (3α,5α)-3-hydroxy-pregnan-20-one (3α,5α-THP, allopregnanolone) is decreased in various brain regions of C57BL/6J mice following exposure to an acute stressor or chronic intermittent ethanol (CIE) exposure and withdrawal. It is well established that there are complex interactions between stress and ethanol drinking, with mixed literature regarding the effects of stress on ethanol intake. However, there is little research examining how chronic ethanol exposure alters stress responses. The present work examined the impact of CIE exposure and withdrawal on changes in brain levels of 3α,5α-THP, as well as hormonal and behavioral responses to forced swim stress (FSS). Adult male C57BL/6J mice were exposed to four cycles of CIE to induce ethanol dependence. Following 8 h or 72 h withdrawal, mice were subjected to FSS for 10 min, and 50 min later brains were collected for immunohistochemical analysis of cellular 3α,5α-THP. Behavioral and circulating corticosterone responses to FSS were quantified. Following 8 h withdrawal, ethanol exposure potentiated the corticosterone response to FSS. Following 72 h withdrawal, this difference was no longer observed. Following 8 h withdrawal, stress-exposed mice showed no differences in immobility, swimming or struggling behavior. However, following 72 h withdrawal, ethanol-exposed mice showed less immobility and greater swimming behavior compared to air-exposed mice. Interestingly, cellular 3α,5α-THP levels were increased in the lateral amygdala 8 h and 72 h post-withdrawal in stressed ethanol-exposed mice compared to ethanol-exposed/non-stressed mice. In the paraventricular nucleus of the hypothalamus, stress exposure decreased 3α,5α-THP levels compared to controls following 72 h withdrawal, but no differences were observed 8 h post-withdrawal. There were no differences in cellular 3α,5α-THP levels in the nucleus accumbens shell at either withdrawal time point. These data suggest that there are different mechanisms mediating hormonal, behavioral, and brain responses to stress following CIE exposure. The lateral amygdala appears to be an extremely sensitive brain region exhibiting changes in cellular 3α,5α-THP levels following CIE and exposure to swim stress. It is likely that these changes in cellular 3α,5α-THP levels in the lateral amygdala contribute to the behavioral effects observed following 72 h withdrawal.

## Introduction

Chronic intermittent ethanol (CIE) exposure and withdrawal produces ethanol dependence and increases subsequent ethanol consumption in C57BL/6J mice (Becker and Lopez, 2004; Griffin et al., 2009; John et al., 2012). One of the most important aspects of the CIE model is the repeated withdrawal that occurs, which is responsible for the sustained increases in ethanol consumption (Lopez and Becker, 2005). Furthermore, CIE exposure blunts hypothalamic–pituitary–adrenal (HPA) axis activation over repeated ethanol exposure, which would result in long-lasting changes in behavioral responsivity to stressors (Becker, 2012).

The 5α-reduced pregnane neurosteroid, (3α,5α)-3-hydroxy-pregnan-20-one (3α,5α-THP; allopregnanolone) is an endogenous neuromodulator synthesized in the brain, adrenals, and gonads that modulates neuronal activity and behavioral output. It is a potent positive allosteric modulator of GABA_A_ receptors that acts with nanomolar potency (Morrow et al., 1987) at known binding sites of GABA_A_ receptors (Hosie et al., 2006) to enhance GABAergic activity, producing similar pharmacological effects as ethanol (Morrow et al., 2004).

Chronic ethanol exposure and withdrawal can be considered a potent stressor that may have an impact on subsequent alcohol and stress responding [reviewed in Heilig et al. (2010)]. Increased reactivity to stress during withdrawal from alcohol can predict relapse in humans and rodents (Lê et al., 1998; Sinha et al., 2000; Sinha, 2008). In humans, alcoholics showed a blunted HPA response to a stressor compared to controls (Adinoff et al., 1990; Lovallo et al., 2000). Repeated ethanol exposure and withdrawal results in persistent adaptations in stress responding in a rat model of ethanol dependence (Zorrilla et al., 2001; Breese et al., 2005). Specifically, ethanol dependence and withdrawal increased anxiety in response to a mild stressor in rats (Zorrilla et al., 2001; Breese et al., 2005). Additionally, altered HPA functionality has been observed following ethanol dependence and withdrawal, manifested as reduced circulating corticosterone levels and increased brain corticosterone levels, an effect that persisted for weeks after cessation of ethanol exposure (Rasmussen et al., 2000; Zorrilla et al., 2001; Little et al., 2008). Together, these data indicate that altered stress responsivity may be related to relapse following ethanol dependence.

Neuroactive steroids alter ethanol intake in a complex manner. Specifically, low doses of 3α,5α-THP increase ethanol self-administration, and higher doses decrease self-administration in C57BL/6J mice (Ford et al., 2005) and in Long-Evans and alcohol preferring P rats (Janak et al., 1998; Morrow et al., 2001). Ethanol consumption is dose-dependently increased by 3α,5α-THP administration in C57BL/6J mice (Sinnott et al., 2002). However, saccharin intake is also increased in C57BL/6J mice following 3α,5α-THP administration, while quinine consumption is not affected. Intracerebroventricular administration of 3α,5α-THP increases ethanol drinking in C57BL/6J mice (Ford et al., 2007). More recently, we have shown pregnenolone decreased ethanol self-administration and increased cerebral 3α,5α-THP levels in alcohol-preferring P rats (Besheer et al., 2010). Furthermore, overexpression of the steroidogenic enzyme cytochrome P450 side chain cleavage in the ventral tegmental area decreases ethanol self-administration, while also increasing 3α,5α-THP levels in the ventral tegmental area in P rats (Cook et al., 2014b). Together, these data indicate that 3α,5α-THP levels can influence drinking behavior in rodent models, but different effects are observed across strains of rodents and doses of neurosteroid.

Ethanol exposure and withdrawal have been shown to regulate 3α,5α-THP levels in the brain. In humans, chronic ethanol exposure and withdrawal decreased 3α,5α-THP levels in human alcoholic patient serum (Romeo et al., 1996). Chronic ethanol exposure and withdrawal decreased hippocampal and cortical 3α,5α-THP levels in male Sprague-Dawley rats (Janis et al., 1998; Cagetti et al., 2004). Finn et al. (2004) have shown that chronic ethanol consumption increased cerebral cortical 3α,5α-THP levels in male C57BL/6 mice. However, using CIE exposure followed by withdrawal, we showed that 3α,5α-THP levels were decreased in several limbic brain regions and increased in CA3 pyramidal cell layer of the hippocampus in C57BL/6J mice. These data indicate the role of 3α,5α-THP may differ in rats and mice, between ethanol exposure models, and following withdrawal from ethanol.

Various stressors have been shown to alter 3α,5α-THP levels in rodent brain. Acute forced swim stress (FSS) increases circulating, cerebral cortical, and hypothalamic levels of 3α,5α-THP and 3α,5α-THDOC, as well as the precursor pregnenolone in various rat strains (Purdy et al., 1991; Barbaccia et al., 1996; Vallée et al., 2000). Furthermore acute ethanol stress also increases 3α,5α-THP levels in rat serum and cerebral cortex (VanDoren et al., 2000), as well as 3α,5α-THP immunohistochemistry (IHC) in rat medial prefrontal cortex, hippocampus, and the paraventricular nucleus of the hypothalamus, but decreases 3α,5α-THP in the nucleus accumbens and the central nucleus of the amygdala (Cook et al., 2014a). In mice, acute FSS decreased 3α,5α-THP immunoreactivity in the lateral amygdala, nucleus accumbens shell, and in the paraventricular nucleus of the hypothalamus (Maldonado-Devincci et al., 2014a). Acute ethanol stress did not alter 3α,5α-THP levels in the cortex or hippocampus in C57BL/6J mice (Porcu et al., 2014). These data indicate there are differences in stress regulation of 3α,5α-THP levels in rats and mice.

We have previously shown that CIE exposure or acute exposure to FSS decrease 3α,5α-THP levels in discrete regions of the extended amygdala (Maldonado-Devincci et al., 2014a,b). Additionally, stress exposure during withdrawal can precipitate relapse to alcohol (Lê et al., 1998). Therefore, it is important to understand the complex changes in 3α,5α-THP levels during withdrawal in response to stress, given this is an extremely vulnerable period in which exposure to stress would likely precipitate relapse to heavy drinking. We investigated how CIE exposure alters behavioral, hormonal, and neurosteroid responses to FSS during ethanol withdrawal in C57BL/6J mice.

## Materials and Methods

### Subjects

Adult male C57BL/6J mice (*n* = 10–13/group) were obtained from Jackson Laboratories (Bar Harbor, ME, USA) at 7–8 weeks of age. Animals were between 9–11 weeks of age at the beginning of the experiment. All mice were allowed to acclimate to the colony before initial exposure to vapor inhalation chambers. Animals were group-housed 5/cage with free access to food and water. All mice were maintained in a temperature (23 ± 0.06°C) and humidity (40 ± 10%) controlled room with lights on from 0700–1900 h. Animal care followed National Institutes of Health Guidelines under University of North Carolina at Chapel Hill Institutional Animal Care and Use Committee approved protocols.

### Apparatus

Glass cylinders (Wholesale Flowers and Supplies; 20 cm diameter, 40 cm height) were used as the swim test apparatus. The glass cylinders were filled with water (23–25°C) to approximately 20 cm in depth. Cylinders were separated by a visual barrier.

### Chronic Intermittent Vapor Inhalation Chamber Exposure

Within 1 week of arrival to the animal vivarium, all mice were subjected to mild restraint for blood collection of approximately 20 μl from the submandibular space for assessment of baseline corticosterone levels. Specifically, mice were mildly restrained, poked in the cheek pouch with a lancet (Medipoint, Inc., Mineola, NY, USA), 20 μl blood collected from the submandibular space, and mild pressure applied to the wound area to promote blood clotting. No anesthesia is required for this method of blood collection.

Mice were exposed to repeated intermittent ethanol exposure or air for four cycles over 4 weeks as described below. Mice were transported from the colony to the procedure room at approximately 1630 h. Animals were weighed and administered an intraperitoneal injection (0.02 ml/g) of the alcohol dehydrogenase inhibitor pyrazole (1 mmol/kg) combined with saline for control animals or combined with 1.6 g/kg ethanol (8% w/v) and immediately placed in the inhalation chambers (La Jolla Alcohol Research Inc., San Diego, CA, USA). Mice remained in the vapor inhalation chambers for 16 h overnight with room air (control group) or volatized ethanol (ethanol group) delivered to the chambers at a rate of 10 liters/min. These procedures were designed to maintain blood ethanol concentrations (BECs) at 150–250 mg/dl throughout the exposure period. Ethanol (95%) was volatilized by passing air through an air stone (gas diffuser) submerged in ethanol. The following morning, at 0900 h, mice were removed from the vapor inhalation chamber and returned to the home cage for 8 h. One cycle consisted of four 16 h exposures. Following each exposure cycle, mice were returned to the home cage and colony and remained undisturbed for 3 days.

On the morning following initial exposure to the vapor inhalation chamber and following the third day of each cycle, mice were removed from the chamber and 20 μL of blood was collected from the submandibular space for BEC assessment (Table 1). BECs were analyzed by gas chromatography with 10 μL of blood diluted in 375 μL of ddH_2_O and 500 mg of NaCl in a sealed 12 × 75 test tube. The sample was volatized in a water bath set at 55°C and 1.5 mL of volatized sample was removed from the tube and injected.

**Table 1:** **Blood ethanol concentrations (mg/dl) across CIE cycles**.

Cycle	8 h Post-withdrawal	72 h Post-withdrawal
1	171.8 ± 9.8	242.5 ± 13.0**
2	209.0 ± 8.5	179.1 ± 8.7
3	182.3 ± 6.6	185.9 ± 8.8
4	206.5 ± 9.5	204.5 ± 12.9

Data are presented as mean blood ethanol concentration (mg/dl) ± SEM across CIE exposure cycles for each experiment. **p < 0.0001 72 h withdrawal greater than 8 h.

Mice were exposed to four cycles of CIE vapor exposure and subsequently underwent withdrawal from ethanol for 8 h (Experiment 1) or 72 h (Experiment 2) withdrawal.

### Forced Swim Stress

On the day of the FSS exposure, all mice were transported to the procedure room to acclimate for a minimum of 60 min. Control animals were maintained in a room separate from the procedure room where mice were exposed to the forced swim test. The FSS occurred between 1500–1700 h for Experiment 1 (8 h withdrawal) or between 0900–1100 for Experiment 2 (72 h withdrawal).

At the beginning of the swim stress session, mice were removed from the home cage, weighed and lowered into the apparatus for a 10 min swim session. Following the swim session, the mouse was removed from the swim tank, lightly dried, and 20 μl of blood was collected from the submandibular space for assessment of stress-induced corticosterone levels. The animal was then returned to the home cage and placed on a warming pad for 15–20 min. Immobility, swimming, and struggling were assessed by experimenter-manual scoring after the session, where the experimenter was blind to the condition of the mouse. Immobility was defined as the mouse remaining completely still or with minimal involuntary movements required to remain afloat. Swimming was defined as relatively calm paddling, including movement of all four legs, or just the two hind legs. Struggling was defined as vigorous swimming, thrashing, and/or climbing in an attempt to escape, which usually subsided by the first three minutes after presentation to the inescapable swim tank. After each swim session, the cylinders were emptied, cleaned, and refilled with fresh water for the next subject. The mice remained in the home cage for 50 min.

Serum blood samples were centrifuged at 1730 g for 15 min at 4°C. Serum was collected and frozen at −80°C until analysis. Serum corticosterone levels were analyzed using a radioimmunoassay kit according to manufacturer’s instructions (MP Biomedicals, Orangeburg, NY, USA).

### Tissue Processing and Immunohistochemistry

IHC was performed on free-floating sections (3–5 sections/animal/brain region) using an affinity purified 3α,5α-THP sheep antibody, that has been previously characterized by radioimmunoassay (Janis et al., 1998; VanDoren et al., 2000) and IHC (Cook et al., 2014a). This antibody labels both 3α,5α-THP and the intermediate 3α-hydroxyprogesterone that also has GABAergic properties and is found in brain. As previously described, sections for 3,3′-diaminobenzidine (DAB, Sigma-Aldrich, St. Louis, MO, USA) were processed identically to recently published methods (Cook et al., 2014a), except that sections were incubated in a rabbit-anti-sheep biotinylated secondary antibody (1:200, Vector Laboratories, Burlingame, CA, USA) that was preabsorbed in 2% mouse serum (Sigma-Aldrich; Maldonado-Devincci et al., 2014a,b).

### Immunohistochemical Analyses

Brain region immunoreactivity was visualized with an Olympus CX41 light microscope (Olympus America, Center Valley, PA, USA), images were captured with a digital camera (Regita model, QImaging, Burnaby, BC, USA), and analyzed using Bioquant (Nashville, TN, USA) image analysis to obtain linear integrated optical density for immunoreactivity assessment. The microscope, camera, and software were background corrected to eliminate non-specific labeling and normalized to preset light levels to ensure fidelity of data acquisition. For each brain region, a threshold is selected based on positive cellular staining of cell bodies. This threshold is then applied to each image and pixel density is quantified. Positive pixel count of immunoreactivity was quantified from a circumscribed field, delineated as a brain region, divided by the area of the region in square millimeters, and expressed as pixels/mm^2^. Data from 4–5 alternate sequential sections per animal per brain region from both hemispheres were used to average one value per mouse. If tissue damage from IHC processing resulted in fewer than three sections/brain region, the mouse was eliminated from analyses. The experimenter was blind to the condition of each animal when analyses were conducted. Matched sections were used for each brain region. Brain regions analyzed included nucleus accumbens shell (+1.42 to +0.98 AP), paraventricular hypothalamic nucleus (−0.58 to −1.06 AP), and lateral amygdala (−1.22 to −1.58 AP). All coordinates were expressed relative to bregma in the Mouse Brain Atlas in Stereotaxic Coordinates (Franklin and Paxinos, 2007).

### Statistical Analysis

All data were analyzed separately for each experiment. Corticosterone data were transformed as a difference score (Post Stress—Baseline) and analyzed using a two-way between subjects design ANOVA with Exposure (Air, Ethanol) and Stress (No Stress, Stress) as factors. Behavioral data were analyzed as a two-way mixed model design ANOVA with Exposure (Air, Ethanol) as a between subjects factor and Behavior (Immobile, Swimming, and Struggling) as a repeated measure. Immunohistochemical data were transformed and expressed as percent control (air/non-stressed group) values and analyzed using a two-way between subjects design ANOVA with Exposure (Air, Ethanol) and Stress (No Stress, Stress) as factors. In the presence of significant main effects or interactions, Fisher’s planned least significant difference (PLSD) test was conducted to isolate effects. The level of significance was set to a α level ≤0.05.

## Results

### Blood Ethanol Concentrations Across CIE Cycles

In general, mice were exposed to comparable ethanol concentrations (>170 mg/dl) across the four CIE cycles (Table 1). Mice exposed to ethanol in the 8 h post-withdrawal experiment had lower BECs compared to those in the 72 h post-withdrawal experiment during the first cycle of CIE exposure (*t*_(252)_ = 4.5, *p* < 0.0001). However, by the second cycle through the end of the experiment comparable BECs were observed between the groups (Table 1, Cycle by Withdrawal Time Point interaction [*F*_(3,189)_ = 10.7, *p* < 0.0001] and a significant main effect of Cycle [*F*_(3,89)_ = 2.8, *p* < 0.05]).

### Corticosterone Response to Forced Swim Stress

**Figure 1:**
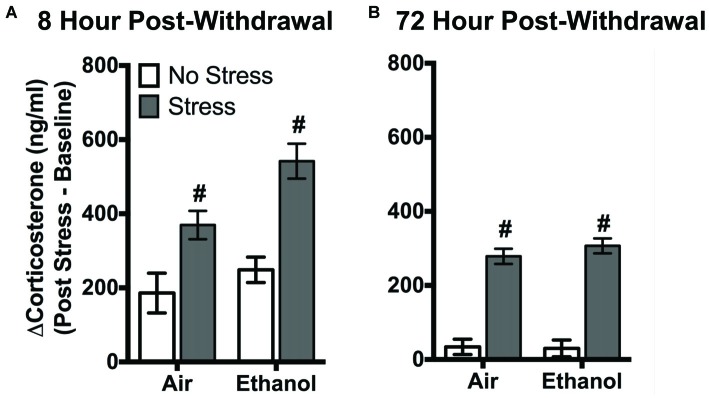
**Effects of chronic intermittent ethanol (CIE) or air exposure on circulating corticosterone (ng/ml) levels following (A) 8 h or (B) 72 h post-withdrawal in non-stressed (clear bars) or stressed mice (gray bars).** Data are depicted as mean difference between post-stress and baseline corticosterone levels ±SEM. ^#^Indicates main effect of Stress (*p* < 0.0001).

FSS following four cycles of CIE altered circulating corticosterone levels in air-exposed and ethanol-exposed mice 8 h and 72 h post-withdrawal (Figure 1). Following 8 h withdrawal (Figure 1A), exposure to FSS increased circulating corticosterone levels in both air-exposed (57.1 ± 11.4%) and ethanol-exposed (115.6 ± 15.1%) mice compared to air-exposed/non-stressed mice (main effect of Stress [*F*_(1,40)_ = 27.11, *p* < 0.0001]). In general, ethanol-exposed mice showed greater corticosterone levels in response to FSS compared to air-exposed mice (main effect of Exposure [*F*_(1,40)_ = 6.60, *p* < 0.05]). Following 72 h withdrawal (Figure 1B), FSS increased circulating corticosterone levels similarly in air-exposed (94.9 ± 22.2%) and ethanol exposed (89.5 ± 10.7%) mice compared to air-exposed/non-stressed mice (main effect of Stress [*F*_(1,47)_ = 153.6, *p* < 0.0001]).

### Behavioral Response to Forced Swim Stress

**Figure 2:**
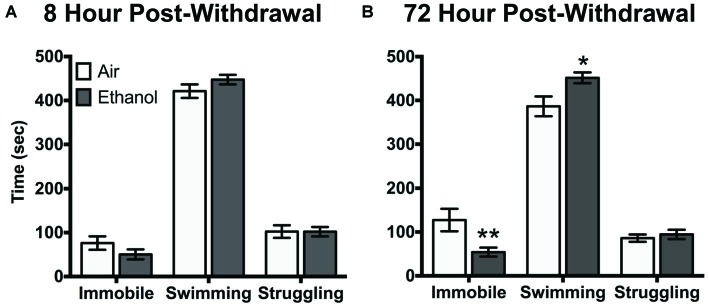
**Behavioral response to forced swim stress (FSS) following (A) 8 h or (B) 72 h withdrawal in air-exposed (clear bars) or ethanol-exposed mice (gray bars).** Data are depicted as mean time engaged in each behavior ±SEM. *p < 0.05 compared to respective Air-Stress group. **p < 0.005 compared to respective Air-Stress group.

Air-exposed and ethanol-exposed mice showed differences in their behavioral response to FSS at 72 h post-withdrawal. Following 8 h withdrawal, mice exposed to FSS showed similar levels of immobility, swimming, and struggling behavior, regardless of exposure to air or ethanol (Figure 2A). However, following 72 h post-withdrawal, air-exposed and ethanol-exposed mice showed behavioral differences in immobility and swimming (Behavior by Exposure interaction [*F*_(2,48)_ = 6.4, *p* < 0.005]). Specifically, ethanol exposed mice showed less immobility (*t*_(72)_ = 3.3, *p* < 0.005) and more swimming (*t*_(72)_ = 2.9, *p* < 0.05) behavior compared to air-exposed mice (Figure 2B). There were no differences in struggling behavior between air-exposed and ethanol-exposed mice in either experiment.

### Chronic Intermittent Ethanol Exposure Alters Brain 3α,5α-THP Levels Following Forced Swim Stress

**Figure 3:**
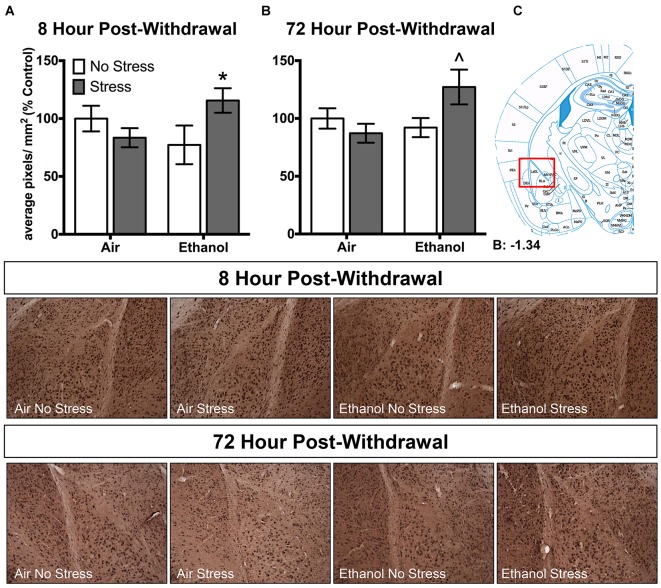
**Effects of CIE or air exposure on 3α,5α-THP immunoreactivity in the lateral amygdala following (A) 8 h or (B) 72 h post-withdrawal in non-stressed (clear bars) or stressed mice (gray bars).** Data depicted are mean positive pixels/mm^2^ expressed as percent control ±SEM. **(C)** Red box indicates coordinates relative to bregma depicted in photomicrographs. Representative photomicrographs (10x) of 3α,5α-THP immunoreactivity 72 h post-withdrawal following CIE and/or stress exposure. *p < 0.05 compared to Ethanol-No Stress group; ^∧^*p* < 0.05 compared to Ethanol-No Stress and Air-Stress group.

Ethanol exposure altered 3α,5α-THP levels in response to FSS in a unique manner in the lateral amygdala at both 8 h and 72 h post-withdrawal (Figure 3). Following 8 h withdrawal (Figure 3A), ethanol-exposed mice that were subjected to FSS showed elevated 3α,5α-THP levels compared to ethanol-exposed/non-stressed mice [*t*_(33)_ = 2.2, *p* < 0.05] as supported by an Exposure by Stress interaction [*F*_(1,33)_ = 5.2, *p* < 0.05]. In contrast, FSS did not alter 3α,5α-THP levels in the air-exposed condition. Similarly, 72 h post-withdrawal (Figure 3B), ethanol-exposed mice that were subjected to FSS showed elevated 3α,5α-THP levels compared to ethanol-exposed/non-stressed mice [*t*_(46)_ = 2.4, *p* < 0.05] and compared to air-exposed/stressed mice [*t*_(46)_ = 2.6, *p* < 0.05] as supported by an Exposure by Stress interaction [*F*_(1,46)_ = 5.1, *p* < 0.05]. No effect of FSS was observed in the air-exposed mice.

**Figure 4:**
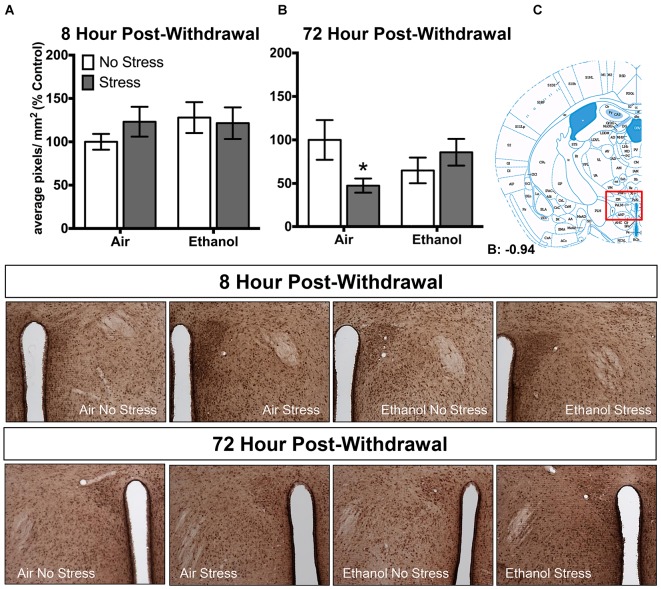
**Effects of CIE or air exposure on 3α,5α-THP immunoreactivity in the paraventricular nucleus of the hypothalamus following (A) 8 h or (B) 72 h post-withdrawal in non-stressed (clear bars) or stressed mice (gray bars).** Data depicted are mean positive pixels/mm^2^ expressed as percent control ±SEM. **(C)** Red box indicates coordinates relative to bregma depicted in photomicrographs. Representative photomicrographs (10x) of 3α,5α-THP immunoreactivity 72 h post-withdrawal following CIE and/or stress exposure. *p < 0.05 compared to Air-No Stress group.

In the paraventricular nucleus of the hypothalamus, no changes in 3α,5α-THP levels were observed following 8 h withdrawal in either the control or FSS condition (Figure 4A). However, 72 h post-withdrawal (Figure 4B), FSS decreased 3α,5α-THP levels in the in air-exposed/stressed mice compared to air-exposed/non-stressed mice [*t*_(40)_ = 2.3, *p* < 0.05] as supported by an Exposure by Stress interaction [*F*_(1,40)_ = 5.4, *p* < 0.05]. When ethanol-exposed mice were stressed, this combination prevented the stress-induced reduction in 3α,5α-THP levels in these mice.

**Figure 5:**
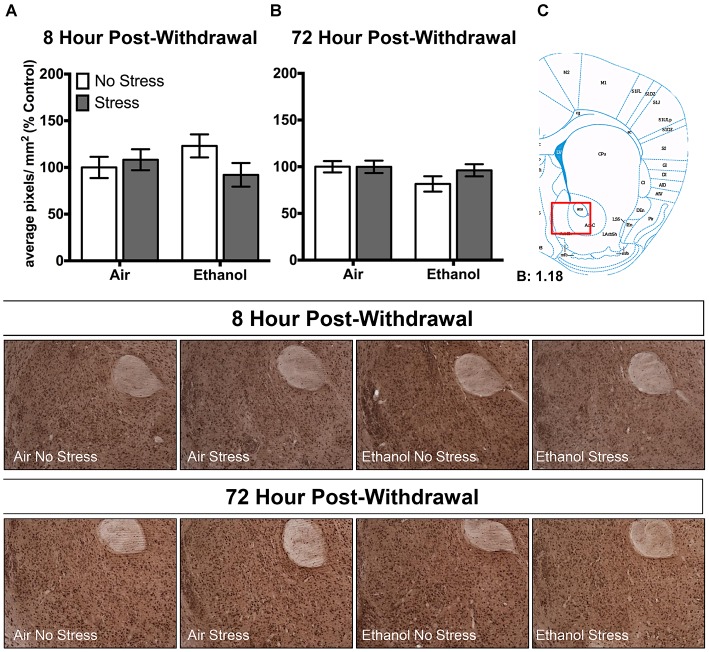
**Effects of CIE or air exposure on 3α,5α-THP immunoreactivity in the nucleus accumbens shell following (A) 8 h or (B) 72 h post-withdrawal in non-stressed (clear bars) or stressed mice (gray bars).** Data depicted are mean positive pixels/mm^2^ expressed as percent control ±SEM. **(C)** Red box indicates coordinates relative to bregma depicted in photomicrographs. Representative photomicrographs (10x) of 3α,5α-THP immunoreactivity 72 h post-withdrawal following CIE and/or stress exposure.

In the nucleus accumbens shell, there were no changes in 3α,5α-THP levels following CIE or FSS exposure following 8 h (Figure 5A) or 72 h (Figure 5B) withdrawal.

## Discussion

Overall, this work illustrates that CIE alters the brain and behavioral stress response in a time-dependent manner. Specifically, stress increased 3α,5α-THP levels following CIE exposure at both 8 h and 72 h withdrawal in the lateral amygdala, while stress had no effect on 3α,5α-THP levels in the air-exposed control animals. Behaviorally, an escalation of swimming behavior coupled to less immobility was observed in CIE-exposed mice following 72 h withdrawal, with no change observed following 8 h withdrawal. To our knowledge, these are the first data to show that a history of CIE exposure alters the 3α,5α-THP response to FSS challenge in C57BL/6J mice. Further, we show an association between elevated 3α,5α-THP levels in the lateral amygdala and increased swimming behavior, consistent with increased reactivity to stress after CIE exposure. The lateral amygdala appears to be an extremely sensitive brain region, exhibiting changes in cellular 3α,5α-THP levels following CIE and exposure to swim stress.

Chronic ethanol exposure and withdrawal can be considered a potent stressor that may have an impact on subsequent alcohol and stress responding [reviewed in Heilig et al. (2010)]. Increased reactivity to stress during withdrawal from alcohol can predict relapse in humans and rodents (Lê et al., 1998; Sinha et al., 2000; Sinha, 2008). Previous studies in mice have suggested that stress can also produce a paradoxical excitatory effect of the GABAergic neurosteroid 3α,5α-THDOC in C57BL/6J mice (Sarkar et al., 2011). Acute restraint stress induced a collapse of the chloride gradient, changing the activity of GABA from inhibitory to excitatory in corticotropin releasing factor neurons in the paraventricular nucleus of the hypothalamus (Sarkar et al., 2011). GABAergic neuroactive steroids are reported to be necessary to mount the appropriate stress response (Sarkar et al., 2011). Thus, it is possible that the increase in 3α,5α-THP levels in the amygdala are related to the exaggerated stress response of increased swimming and decreased immobility in the mice. Indeed, Wistar rats locally injected with 3α,5α-THP in the septum or hippocampus showed lower immobility in the forced swim test compared to vehicle-injected rats (Rodríguez-Landa et al., 2009). Interestingly, the reported relationship between higher 3α,5α-THP levels and decreased immobility is similar to the effect of CIE and stress exposure in C57BL/6J mice in the present work.

Sommer et al. (2008) have posited that intra-amygdala systems mediate increased stress sensitivity in the post-dependent state in Wistar rats following ethanol dependence, which is expressed following a stress challenge. Recently, it has been shown that rats exposed to chronic ethanol and swim stress show greater neuronal activation in the medial and capsular aspects of the CeA compared to controls (Retson et al., 2015). Additionally, exposure to FSS has been suggested to be a more robust stressor than ethanol exposure by increasing neuronal activation in the CeA to a greater extent compared to ethanol-exposure alone (Retson et al., 2016). These authors propose chronic ethanol exposure alters the response to stress by altering neuronal activation in the CeA (Retson et al., 2015). This idea is consistent with the elevated 3α,5α-THP levels observed in the lateral amygdala in ethanol-exposed mice subjected to FSS. Previously we have conducted co-localization analysis and observed co-localization of 3α,5α-THP-positively labeled neurons with both glutamatergic (VGlut1) and GABAergic (VGAT) markers in the lateral amygdala. However, we have not assessed the phenotype of the neurons that exhibit the change in 3α,5α-THP levels. This will be addressed in future studies.

The lateral amygdala serves as a sensory information gating region that receives input from many brain regions and projects to other subregions of the amygdala and reciprocally back to input regions (Pitkanen et al., 1997; Faber et al., 2001). The combination of CIE exposure and FSS may alter the sensory information gating of the lateral amygdala to other amygdalar subregions and to output regions, including the paraventricular nucleus of the hypothalamus, medial prefrontal cortex and the hippocampus, resulting in changes to input/output signals of excitation and inhibition. The increase in 3α,5α-THP levels following CIE exposure and stress challenge in the present work may be associated with altered neuronal activation (Tokuda et al., 2011). In the present work the lateral amygdala appears to be an extremely susceptible brain region with alterations in 3α,5α-THP levels following both CIE and FSS exposure.

Changes in behavioral responsivity to stress have also been observed in rat models of ethanol dependence and stress exposure. Our results differ from other studies in rats where chronic ethanol exposure produces a blunted response to stress. When ethanol-dependent Wistar rats were subsequently exposed to FSS, they showed a potentiation in ethanol drinking compared to nondependent rats (Sommer et al., 2008). However, when ethanol-dependent Sprague-Dawley rats are still under the influence of ethanol and subjected to FSS, they showed less swimming and more immobility compared to control rats (Retson et al., 2015). These data suggest that behavioral responses during withdrawal are different than those that occur when the animals are still under the influence of ethanol. The mice in the present work responded to stress by expending more energy through increased swimming and less immobility compared to air-exposed mice.

Prince and Anisman (1984) suggest that engaging in more floating behavior may be an adaptive coping strategy to conserve energy. Thus, the decreased floating behavior that was observed in the chronically CIE-exposed mice in the present work may represent a maladaptive response to FSS. Other groups have observed similar adaptations to chronic stress. In chronically stressed mice, i.e., chronic footshock stress, challenged with an acute stressor (footshock or restraint) on the test day, there was decreased floating in the forced swim test compared to chronically footshock stressed mice that did not receive the acute stress challenge (Dunn and Swiergiel, 2008). In another study, CD-1 mice that were chronically exposed to footshock showed increased immobility in the forced swim test compared to controls (Swiergiel et al., 2008). However, acute footshock challenge after chronic exposure to footshock decreased immobility compared to chronically shocked mice that did not receive the acute footshock challenge (Swiergiel et al., 2008). Swiergiel et al. (2008) suggest that exposure to an acute stressor after a chronic stressor reverses the behavioral change observed in the forced swim test. Furthermore, there are innate differences between rats and mice and their stress responses. Historically the rattus norvegicus, the ancestor of the common laboratory rat, is well adapted to a semi-aquatic environment. The ancestor of the common laboratory mouse, mus musculus adapted to a dry environment. Therefore, more swimming would be considered the adaptive stress response in the mouse.

Changes in corticosterone levels have been observed following ethanol dependence and withdrawal. Specifically chronic exposure to ethanol reduced circulating corticosterone levels for weeks after cessation of ethanol exposure (Rasmussen et al., 2000; Zorrilla et al., 2001). Wistar rats showed decreased corticosterone levels following 1 day withdrawal from ethanol liquid diet compared to pair-fed controls (Zorrilla et al., 2001). Following 1 day withdrawal from liquid diet there were no differences in plasma corticosterone levels and by 72 h post-withdrawal ethanol-exposed Sprague-Dawley rats showed decreased plasma corticosterone levels compared to controls (Rasmussen et al., 2000). C57BL/6 mice exposed to ethanol in their drinking water over 4 weeks and subsequently exposed to ethanol liquid diet over 5 days to induce dependence showed no differences in plasma corticosterone levels compared to isocaloric sucrose-fed mice (Kakihana, 1979). In contrast, following withdrawal from 72 h ethanol vapor exposure AKR/J, Withdrawal Seizure Prone (WSP), and Withdrawal Seizure Resistant (WSR) mice showed elevated corticosterone levels compared to control mice between 0–12 h post-withdrawal, but returned to basal levels by 24 h post-withdrawal (Keith et al., 1983; Beckley et al., 2008; Tanchuck et al., 2009). Similarly, other work in C57BL/6J mice using CIE vapor exposure over 4 days showed elevated corticosterone levels between 4–6 h post-withdrawal, but returned to baseline levels by 4 days post-withdrawal (Healey et al., 2008). Our findings are similar to the work conducted by Kakihana (1979) and may indicate that longer exposure to ethanol may blunt the corticosterone response following ethanol dependence and withdrawal.

Our results also differ from other studies in rats and humans where chronic ethanol exposure produces a blunted HPA response to stress. In humans, alcoholics and controls had similar basal corticosterone levels, but showed a blunted HPA response to a stressor compared to controls (Adinoff et al., 1990; Lovallo et al., 2000). In ethanol-dependent Sprague-Dawley rats that were still intoxicated, no differences in corticosterone levels in response to stress were observed between ethanol-exposed and pair-fed controls (Retson et al., 2015). In the present work, we observed increased corticosterone levels in response to stress in both air-exposed and ethanol-exposed groups at both withdrawal time points. However, since we assessed corticosterone levels immediately upon removal from the swim tank and not at any other time point, we likely did not detect the peak corticosterone response and we cannot determine any changes regarding the temporal profile of the stress response following CIE exposure.

There are several findings that differ from our previous work. Specifically, we showed that CIE exposure decreased 3α,5α-THP levels in the lateral amygdala following both 8 h and 72 h withdrawal (Maldonado-Devincci et al., 2014b). In the present work, we show that CIE exposure tended to decrease 3α,5α-THP levels in the lateral amygdala following 8 h withdrawal (−22.8 ± 20.1%, *p* = 0.13) with no change following 72 h withdrawal (Figure 3). These differences between studies could be attributed to the fact that the non-stressed mice in the current study were removed from their home cage and placed into a clean novel cage for 50 min prior to brain collection. Exposure to a novel cage could be considered a mild stressor (Pace and Spencer, 2005) and therefore may have masked effects of CIE exposure that were expected.

Additionally, we recently showed that acute exposure to FSS decreased 3α,5α-THP levels in the lateral amygdala, paraventricular nucleus of the hypothalamus, and in the nucleus accumbens shell (Maldonado-Devincci et al., 2014a). In the present work, we showed that exposure to FSS decreased 3α,5α-THP levels by 16.6 ± 13.8% in the lateral amygdala (Figure 3A), and produced no changes in the paraventricular nucleus of the hypothalamus (Figure 4A) or in the nucleus accumbens shell (Figure 5A) in the 8 h post-withdrawal experiment. We also show that exposure to FSS decreased 3α,5α-THP levels by 12.8 ± 12.0% in the lateral amygdala (Figure 3B) and by 52.5 ± 24.3% in the paraventricular nucleus of the hypothalamus (Figure 4B) in the 72 h post-withdrawal experiment. It is not entirely clear why the effects of FSS are different between studies, but one critical methodological variable may account for this difference. In the current study, the control mice were chronically exposed to air in the inhalation chambers. Therefore, these mice were treated very differently than the no-stress controls in our previous work (Maldonado-Devincci et al., 2014a). These results suggest that air exposure alone may produce changes in 3α,5α-THP levels in lateral amygdala. Further studies are needed to evaluate this possibility.

The general consensus is that 3α,5α-THP serves to maintain homeostasis in the brain. The present work shows that CIE exposure changes the stress response. Ultimately, chronic exposure to ethanol may change the role of 3α,5α-THP, such that it no longer serves to maintain homeostasis and induces the paradoxical excitatory effect of GABAergic neurosteroids observed in conjunction with acute stress (Sarkar et al., 2011). Thus, the increase in 3α,5α-THP following CIE exposure and stress may increase the excitatory effects on lateral amygdala sensory gating, which likely mediates the maladaptive stress responses observed in the present work. Together, these data indicate that CIE exposure in the C57BL/6J mouse produces a dysregulated stress response.

An alternative effect of CIE exposure and stress challenge could be a change in the steroid biosynthetic pathway. Normally, progesterone is reduced by the biosynthetic enzymes 5α-reductase and 3α-hydroxysteroid dehydrogenase to produce 3α,5α-THP. It is probable that this pathway is altered and progesterone is reduced by CYP21 and CYP11B1 to produce corticosterone instead (Porcu et al., 2014). This hypothesis is supported by recent work showing that acute ethanol administration produced no change in 3α,5α-THP levels, but elevated corticosterone levels were observed in cerebral cortex and hippocampus (Morrow, 2007; Porcu et al., 2014). Thus, corticosterone may be produced instead of 3α,5α-THP, which would further support the hypothesis of the dysregulated stress response following chronic ethanol exposure and stress in the C57BL/6J mouse (Morrow, 2007; Porcu et al., 2014). Both of these possibilities are currently under investigation.

## Author Contributions

AMM-D and ALM were responsible for the study concept. AMM-D and TKO performed the ethanol exposure paradigm. AMM-D, TKO, DHM and REM performed the FSS exposure. AMM-D, AK-P, REM and DHM conducted IHC protocols. AMM-D and AK-P collected immunohistochemical data. AMM-D and REM measured BECs and quantified the behavioral data. AMM-D and TKO quantified the corticosterone data. AMM-D analyzed the IHC, behavioral and corticosterone data, interpreted the results, and drafted the manuscript. All authors critically reviewed the content of the manuscript and approve the final version for publication.

## Funding

This research was supported by the NIAAA INIA U01-AA020935 (ALM).

## Conflict of Interest Statement

The authors declare that the research was conducted in the absence of any commercial or financial relationships that could be construed as a potential conflict of interest.
